# Are health facilities well equipped to provide basic quality childbirth services under the free maternal health policy? Findings from rural Northern Ghana

**DOI:** 10.1186/s12913-018-3787-1

**Published:** 2018-12-12

**Authors:** Philip Ayizem Dalinjong, Alex Y. Wang, Caroline S. E. Homer

**Affiliations:** Faculty of Health, University of Technology Sydney, Sydney, New South Wales Australia

**Keywords:** Availability, Maternal health services, Basic inputs, Infrastructure, Privacy, Childbirth, Quality care, Free maternal health policy, National Health Insurance, Ghana

## Abstract

**Background:**

Basic inputs and infrastructure including drugs, supplies, equipment, water and electricity are required for the provision of quality care. In the era of the free maternal health policy in Ghana, it is unclear if such basic inputs are readily accessible in health facilities. The study aimed to assess the availability of basic inputs including drugs, supplies, equipment and emergency transport in health facilities. Women and health providers’ views on privacy and satisfaction with quality of care were also assessed.

**Methods:**

The study used a convergent parallel mixed methods in one rural municipality in Ghana, Kassena-Nankana. A survey among facilities (*n* = 14) was done. Another survey was carried out among women who gave birth in health facilities only (*n* = 353). A qualitative component involved focus group discussions (FGDs) with women (*n* = 10) and in-depth interviews (IDIs) with midwives and nurses (*n* = 25). Data were analysed using descriptive statistics for the quantitative study, while the qualitative data were recorded, transcribed, read and coded using themes.

**Results:**

The survey showed that only two (14%) out of fourteen facilities had clean water, and five (36%) had electricity. Emergency transport for referrals was available in only one (7%) facility. Basic drugs, supplies, equipment and infrastructure especially physical space were inadequate. Rooms used for childbirth in some facilities were small and used for multiple purposes. Eighty-nine percent (*n* = 314) of women reported lack of privacy during childbirth and this was confirmed in the IDIs. Despite this, 77% of women (*n* = 272) were very satisfied or satisfied with quality of care for childbirth which was supported in the FGDs. Reasons for women’s satisfaction included the availability of midwives to provide childbirth services and to have follow-up homes visits. Some midwives were seen to be patient and empathetic. Providers were not satisfied due to health system challenges.

**Conclusion:**

Government should dedicate more resources to the provision of essential inputs for CHPS compounds providing maternal health services. Health management committees should also endeavour to play an active role in the management of health facilities to ensure efficiency and accountability. These would improve quality service provision and usage, helping to achieve universal health coverage.

**Electronic supplementary material:**

The online version of this article (10.1186/s12913-018-3787-1) contains supplementary material, which is available to authorized users.

## Background

The availability of infrastructure, utilities, drugs, supplies and equipment provide the enabling environment for health facilities and health providers to function effectively. In health service delivery, utilities such as water and electricity are vital for the provision of quality care [1–4]. The World Health Organization (WHO) recommends the availability of water, sanitation and hygiene (WASH) services in health facilities for the provision of quality, people-centered care [5–8]. WASH increases health provider’s morale, efficiency, trust and the use of health services, including a reduction in cost of service delivery [7]. Electricity in particular, allows for the effective operations of the cold chain system [1, 7] and for lighting as well.

Nevertheless, a study in 54 low- and middle-income countries reported that 38 and 19% of health facilities respectively, lacked access to clean water and sanitation, while 35% did not have water and soap for hand washing [7]. In sub-Saharan Africa, 50% of health facilities do not have clean water [6, 7]. Another review showed that 26% of health facilities in eleven sub-Saharan African countries did not have electricity [3]. Even for hospitals which are meant to be points of referral for lower level health facilities, aggregate data from the Service Provision Assessments and Demographic and Health Surveys for five sub-Saharan African countries (Ghana, Kenya, Tanzania, Rwanda and Uganda) demonstrate that only 20 to 46% of these health facilities had reliable running water and electricity [9]. Health facilities located in rural communities are worse off in the provision of basic essential inputs compared to bigger health facilities in urban areas [7].

Two of WHO’s quality standards (standards 5 and 8) have also emphasised the importance of basic drugs, supplies, equipment, privacy and confidentiality in health facilities for improved maternal and newborn health [8]. The absence of such basic essential inputs hamper the provision and use of maternal health services [10–13]. For example, a study in an urban hospital in Tamale, Ghana, revealed the lack of reliable water and electricity supply, essential drugs, manual vacuum aspirators and surgical gloves which negatively impacted the provision of childbirth services [14]. Likewise, the lack of privacy in health facilities in South Central Ethiopia, deterred women from utilising health facilities for childbirth [15]. It is therefore important to examine the availability of basic essential inputs such as water and electricity, basic drugs, supplies, and equipment for service delivery to better understand the drivers of quality of care [8, 16].

In Ghana, a free maternal health policy was implemented in July 2008, under the National Health Insurance Scheme. The policy provides free of cost maternal health services to Ghanaian women. It seeks to promote the use of skilled attendance for the reduction of maternal and newborn deaths. There are limited studies assessing the availability of basic essential inputs in health facilities for childbirth and how these impacted on women and health providers’ perception of quality care. The Ghanaian study in Tamale did not include health centres and Community-based Health Planning and Services (CHPS) compounds. This study aimed to assess the availability of basic essential inputs including drugs, supplies and equipment in health facilities offering childbirth services in one rural area in Ghana. Women and health provider’s perception of privacy and satisfaction with quality of care at childbirth were also assessed. This paper forms part of a wider research exploring the affordability, availability, acceptability and quality of maternal health services during pregnancy and childbirth under the free maternal health policy in rural Northern Ghana [17, 18].

## Methods

### Study area

A cross sectional study was carried out in the Kassena-Nankana municipality, a rural poor settings located in the Upper East Region of Northern Ghana, with the capital town as Navrongo. The study was carried out from March – August, 2016. The population of the study area was about 109, 944 representing 10.5% of the total population of the region [19]. About 72.7% of the people live in rural areas and 96.1% of households engage in crop farming including poultry rearing [19]. An estimated 44.8% of the population aged 12 years and above are married, while 56.3% are literate (11 years and beyond) [19]. Skilled attendance at childbirth was estimated to be 67% in the municipality [20]. At the time of the study, 13 of the 14 health facilities studied were publicly owned. The studied health facilities comprise the main district hospital, 2 health centres, and 11 Community-based Health Planning and Services (CHPS compounds). The CHPS compounds are the lowest level of health facilities providing services including maternal health in distant and remote communities [21]. The first CHPS compounds were experimented in the Kassena-Nankana area, before their scale up nationally.

### Study design and processes

The study was a convergent parallel mixed methods, using quantitative and qualitative data collection methods [22]. The collection, analysis and interpretation of the quantitative and qualitative data were carried out in a parallel form [22]. The results of the quantitative and qualitative studies were however, merged to answer questions on availability of basic essential inputs including basic drugs, supplies, and equipment as well as perception of privacy and quality of care.

The formula proposed by Gorstein et al. was used to determine the sample size for a proportion in a single cross-sectional survey for the quantitative component [23], recruiting 353 women who gave birth in health facilities (Additional file 1). The overall sampling procedure has been reported in [18]. Table 1 summarised the study design and processes.

**Table 1:** Summary of study design and processes

Study design	Methods & numbers	Research instruments	Respondents and processes
Quantitative (Additional files 1 and 2)	Facility survey (14 facilities)	Checklist for availability of basic amenities, equipment, drugs and supplies for childbirth	•Midwives and nurses
			•Survey conducted in same facilities where structured questionnaire were carried out among women
	Survey (353 women who gave birth in health facilities only)	•Structured Questionnaire developed based on literature and in English	•Women who gave birth only in facilities were interviewed
		•Questions were translated into local dialects (Kasem and Nankam) and back, for validity	•Women were recruited after discharge from facilities
		•Questionnaire piloted with women before actual administration	•Interviews took 30–45 min
Qualitative (Additional files 3 and 4)	Focus Groups Discussions (FGDs = 10)	•Interview guide: semi-structured open-ended questions with probes	•Same category of women who gave birth in facilities
		•Guide developed in English and translated into two local dialects (Kasem and Nankam)	•Groups of 5–12 women
		•Language experts carried out front and back translation of the guide to ensure accuracy	•Discussions took place in facilities without providers presence
		•Piloted with women	•Discussions recorded with permission
			•Continuous update of guide with issues emerging from discussions
			•Took about 45–120 min for each discussion
	In-depth Interviews (IDIs = 25)	•Interview guide: semi-structured open-ended questions with probes	•Midwives and nurses
		•Developed in English, but not translated; all providers spoke and understood English	•Interviews took place in private rooms in facilities
		•Piloted with providers	•Interviews recorded with permission
			•Continuous update of guide with issues emerging from interviews
			•Took about 45–120 min for an interview

As indicated, 14 health facilities were surveyed (same facilities where the survey with women took place), on the basis of the availability of at least a midwife to provide basic maternal health services, including childbirth. The use of health facility surveys are important for monitoring the availability of resources, performance, areas requiring improvement as well as the impact of health policies or interventions [16, 24]. A check list was developed and used for the health facility survey (Additional file 2).

Ethical clearance for the study was obtained from the Institutional Review Board of the Navrongo Health Research Centre (approval number NHRCIRB217) and the Human Ethics Review Committee of the University of Technology Sydney (approval number ETH16–0263). All participants were consented using a written consent form. Written permission was also obtained from directors of health services as well as managers of health facilities.

### Study variables

The physical availability of basic essential inputs like clean water and electricity, basic drugs, supplies, and equipment for the provision of quality services were assessed (Additional file 2). The WHO proposed six building blocks to assist analyse health systems and also as points for interventions. These building blocks included: service delivery; health workforce (staff and training); information, medical products, vaccines and technology (drugs, supplies and equipment); financing; leadership and governance [16]. The study was structured around these building blocks, with emphasises on the service delivery block, particularly the section on availability of basic emergency obstetric and newborn care (EmONC) [16, 24].

Women and health providers’ overall satisfaction with quality of care was also assessed (Additional files 3 and 4). Satisfaction for quality of care was assessed as; “very satisfied”, “satisfied”, “normal”, “dissatisfied” and “very dissatisfied”. In addition, the study assessed the availability of privacy in health facilities and other issues relating to childbirth such as physical space. This study did not delve into the availability of staff in general, since the focus was the provision of basic maternal health services including childbirth and thus covered health facilities that had at least a midwife to provide such services.

### Data management and analysis

SurveyCTO Collect v2.10 application was used for the collection of the quantitative data. The SurveyCTO Collect application works on hand-held gadgets allowing for the capture, processing and transport of data for analysis. STATA 14 was used to clean and analyse the data and the findings presented using descriptive statistics.

All audio recordings were transcribed verbatim. Some audio recordings were selected, listened to and compared with the transcripts to ensure accuracy in the transcriptions. Differences between the recordings and transcripts were rectified prior to coding. The transcripts were then reviewed a number of times to help identify emerging themes and sub-themes, which reflected insights of the subjective experiences of women especially. Following the review, codes were assigned to the themes and sub-themes identified in relation to childbirth. The results are presented based on the assigned themes and sub-themes as well as the use of key quotes from the participants.

## Results

### Background characteristics of women and use of different health facility for childbirth

Findings from three hundred and fifty-three (353) women who gave birth in health facilities are presented here. The women had an average age of 27 years. Over half of the women (69%, *n* = 243) reported that they used a different health facility for childbirth from antenatal care. The most cited reason for change of health facility was “referral” (40.2%, *n* = 142) by health providers (Table 2). Health providers’ behaviour, distance and time to health facilities as well as quality of care were not found to be major issues for the change of health facility for childbirth.

**Table 2:** Reasons for use of different health facility for childbirth

Reasons for change of health facility for childbirth	*N* = 353	%
Was referred by health provider	142	40.2
Childbirth service available	19	5.4
Qualified staff available	12	3.4
Quality services available	38	10.8
Good provider behaviour	2	0.7
Short distance and time	8	2.2
Other	22	6.2
NA (used same facility for pregnancy and childbirth)	110	31.1
Total	353	100

*“NA” refers to women who used the same health facility for antenatal care and childbirth and their views captured elsewhere*

### Availability of basic essential inputs for childbirth

Only two (14%) out of the fourteen health facilities had a source of clean water, and five (36%) had electricity (Table 3). Eleven health facilities (79%) had guidelines for the management of pregnancy and childbirth and in nine (64%), staff had refresher training in the last one year preceding the study. Only one health facility (7%) had emergency transport (pick-up vehicle) for the referral of women.

**Table 3:** Availability of basic essential inputs in health facilities

Basic essential inputs (utilities)	*N* = 14 (facilities) Yes *n *(%)
Clean water	2(14%)
Electricity	5(36%)
Emergency transport	1(7%)
Health workforce (guidelines and staff training)	
Guidelines	11(79%)
Staff training	9(64%)
Medical products, vaccines and technology (drugs, supplies, equipment)	
Basic drugs for EmONC	
Antibiotic eye ointment	8(57%)
Injectable oxytocin/ergometrine	9(64%)
Oral antibiotic	14(100%)
Magnesium sulphate	9(64%)
Injectable antibiotic	9(64%)
Intravenous solution and infusion set	14(100%)
Other drugs	
Vitamin K	9(64%)
Basic supplies and equipment	
Skin disinfectant	6(43%)
Cotton and gauze	14(100%)
Gloves	14(100%)
Partograph	14(100%)
Cord clamp	8(57%)
Delivery bed	14(100%)
Examination light	5(36%)
Scissors and blade	9(64%)
Suction apparatus	3(21%)
Needles and syringes	10(71%)
Suture material/needle holder	14(100%)
Forceps	14(100%)
Speculum	12(86%)

Some of the equipment in the health facilities were found to be outdated and inadequate, particularly beds for childbirth and postpartum care. Most CHPS compounds had just one or two beds for childbirth. In situations where two or three women were in labour, some of these women would have to use the floor. In the IDIs, a midwife said:

*“Infrastructure hmmm, that is where I think the challenge is, it is not so much but at least we are dealing with what we have. This labour ward has a 2 bed capacity but sometimes women coming in can be 4-5 and some will be on the floor and sometimes you can even have 3 women pushing at the same time”* (IDIs, midwife).

The IDIs showed that basic drugs and supplies were available, but inadequate in the health facilities. The midwives reported that the quantities of basic drugs and supplies for childbirth especially were insufficient to meet the demand or utilisation. A midwife in a CHPS compound reported:

*“We’ve the basic drugs and supplies, but it is always not enough for us. Most of the times, we get out of stock and have to wait for supplies from the DHMT [District Health Management Team]”* (IDIs, midwife).

### Privacy in health facilities during childbirth

Of the 353 women who gave birth in health facilities, 89% (*n* = 314) reported that there was no privacy during childbirth. Only 11% (*n* = 39) stated that there was privacy. The finding was confirmed in the IDIs that there was no privacy in health facilities at childbirth. A midwife said:

*“So you see that some of the clients will be sitting here; if you’re talking inside and you’re talking louder, some of them may hear what you’re conversing with the other one”* (IDIs, midwife).

The rooms used for childbirth in the smaller health facilities (CHPS compounds) especially, were considered to be small (limited physical space). In some cases, the rooms were used for multiple purposes; that is, abdominal and vaginal examination, childbirth and postpartum care. A nurse reported:

*“…. the labour room is like a cubicle – chop box, that’s where they deliver women, that’s where we do dressing, if we receive an emergency case that’s where we put them……There is no privacy in that room at all”* (IDIs, nurse).

### Women’s overall satisfaction during childbirth

In order of frequency, 77% of the women (*n* = 272) were very satisfied or satisfied with the quality of care at childbirth, 3% (*n* = 11) normal, 20% (*n* = 70) very dissatisfied or dissatisfied. In the FGDs too, most of the women were found to be satisfied with the overall services provided at childbirth. Some midwives were reported to be patient, empathetic and would not shout or insult women. A woman said:

*“I am satisfied because the midwife is patient for us and does not shout at us and if the baby is coming out, she is also always fighting like she is the one in labour just for you to have a safe labour”* (FGDs, woman).

Some other women provided the reason that their midwives were always available, even at night to attend to them, when it was necessary. A woman said:

*“When I was in labour it was in the night but I still told them [family members] to bring me here [health facility] because during ANC the nurse [midwife] treated me well. Even at night, her husband brought her and she came to attend to me here. She struggled before my baby came and she did not insult me talk-less beating me”* (FGDs, woman).

Some midwives were able to do home visits after childbirth to check on the welfare of women and their babies and also to encourage women to continue to visit health facilities. This is the case with CHPS compounds located in the communities and was a source of satisfaction. A woman reported:

*“I am happy, she [midwife] treated me nicely during ANC and after I delivered she still followed me home and visited me about 3 times”* (FGDs, woman).

However, a few of the women in the FGDs said they were not satisfied with the overall quality because they had experienced an inhumane treatment; abandonment, beatings and insults from some midwives and nurses. A woman who gave birth in the district hospital reported that she was beaten by an elderly midwife. A woman described:

*“I delivered at War Memorial [the district hospital]. I had a lot of problems because my baby was coming and I was screaming and an elderly nurse [midwife] came and beat me”* (FGDs, woman).

Another woman said:

*“When I came to deliver she treated me like I was not human, she kept me in the room and left me there so my baby nearly fell off and she still came and was insulting me”* (FGDs, woman).

### Health providers’ overall satisfaction with quality of care for childbirth

Due to challenges with the health system which prevents women from using certain services, health providers indicated that they were not very satisfied with the overall quality of care provided. For instance, a midwife said:

*“The scan is not covered by health insurance and some women always don’t have money but you will really see that they need to take a scan on their pregnancy to be sure of safe delivery, but you cannot do anything to help them so that one if she doesn’t take the scan where is the quality you are providing because that one you don’t know how she is faring but you are agreeing to manage her like that……”* (IDIs, midwife).

But the dedication and commitment to duty on the part of some health providers have provided a degree of satisfaction for quality of care rendered. A nurse explained:

*“Satisfied? Not very satisfied, but satisfied. At least the way I’ve seen the midwife handle them [women], it’s okay; it’s commendable. Sometimes she even goes all the way to the houses to look for them. We don’t have a lot of midwives who will do that. So for her being here, and me coming to meet her, and within just these 3 weeks, it’s very commendable”* (IDIs, nurse).

## Discussion

The study showed that only two (14%) out of the fourteen health facilities had a source of clean water, and five (36%) had power supply. Just one health facility had emergency transport for referrals. Basic drugs, supplies, and equipment including beds were also inadequate for childbirth. Eighty-nine percent (*n* = 314) of the women reported the lack of privacy in health facilities during childbirth. The rooms used for childbirth in CHPS compounds especially, were small in size and used for multiple purposes. Despite these issues, 77% of women (*n* = 272) were very satisfied or satisfied with the quality of care for childbirth. Reasons for women’s reported satisfaction were the availability of midwives to provide childbirth services always and to make follow-up visits to women’s homes after childbirth. Some midwives were also patient, empathetic, and would not shout or insult. Health providers indicated that they were not very satisfied with the quality of care provided.

### Availability of clean water and electricity for childbirth

Two (14%) out of the fourteen health facilities had a source of clean water, and five (36%) had power supply. But studies have shown that the availability of clean water, electricity, telephones, and toilet facilities significantly increases the use of skilled attendance at childbirth [2, 25]. Skilled attendance at childbirth in the study area was 67% [20]. Our finding is not unique. In Nnewi, Nigeria, Nnebue et al. found that clean water, electricity and a refuse disposal system were not available in most of their health facilities [26]. Likewise, an urban hospital study in Ghana, Tamale, revealed that water and electricity were intermittently disrupted and thus affected the smooth provision of health services [14]. The availability of clean water and electricity is critical for ensuring the provision of quality maternal health services [3, 4, 27]. Water is required for maintaining hygiene and sanitation (that is, washing of hands and cleaning of equipment) for the prevention of infections, while electricity is needed for lighting and the refrigeration of blood, vaccines (cold chain system) as well as the sterilisation of basic equipment for childbirth [1, 4]. The absence of clean water and electricity in most of our study health facilities compromise quality of care and an affront to the achievement of universal health coverage and the SDGs.

### Availability of emergency transport for childbirth

Transport is yet another facilitator for the use of maternal health services, especially in times of childbirth. Unfortunately, our study reported emergency transport as available in only one health facility. The reported mode of emergency transport was a pick-up vehicle which lacks comfort and the necessary accessories to support pregnant women. Studies in Africa and Asia have reported similar findings [1, 14, 28, 29]. The absence of emergency transport would not permit women to have timely access to health facilities and services, which may have dire consequences, such as deaths of mothers and neonates. For example, a qualitative study in rural Gambia showed the unavailability of transport as the main factor for increased perinatal deaths [30]. The lack of emergency transport in distant and remote communities is considered one of the reasons why women give birth at home [31]. To reduce maternal and newborn deaths, there is the urgent need to prioritise transport especially for distant and remote communities.

### Inadequate basic drugs, supplies, equipment and physical space

Our study reported the inadequacy of basic drugs, supplies, equipment and physical space for childbirth as reported in other studies. A recent review reported the inadequacy of equipment, drugs and supplies such as gloves and blood as a global phenomenon [32]. A Nigerian study also revealed the inadequacy of equipment, drugs and supplies including physical space for childbirth services [26]. In resource constraint settings especially, there are reported cases of overcrowding in health facilities where women in labour would have to queue for services and share beds with other women during and after childbirth [32]. This is attributed to the lack of staff, physical space and equipment [32]. In some situations, women would have to lie on floors or on bare mattresses soiled with urine, faeces, blood, vomit, and other fluids after childbirth [14, 27, 32]. The inadequacy of basic drugs, supplies, equipment and physical space contravenes the quality statements (statement 8) of the WHO, stipulating for a positive experience for all women in the period of pregnancy, childbirth and postpartum [8].

### Lack of privacy in health facilities during childbirth

Our study recorded lack of privacy in health facilities for women; 89% (*n* = 314) of the women reported the lack of privacy at childbirth and this was confirmed in the IDIs. The lack of privacy for women during childbirth was also reported as a global challenge [32]. The review by Bohren et al. showed the lack of privacy in labour wards during vaginal and abdominal examinations with the unavailability of curtains to separate women from other patients [32]. A recent review in Nigeria showed the lack of privacy where women in labour were attended to, in the presence of several health workers and trainees [33]. Lack of privacy is undignifying and shameful for women. Again, the finding infringes quality statement 5.1 of the WHO canvassing for all women and newborns to have privacy, including respect for their confidentiality during and after childbirth [8].

Lack of privacy has several implications. According to Lothian, privacy during childbirth is important for women as it determines to some extent the outcome of the process [34]. It does not also promote the use of skilled attendance at childbirth. For instance, in South Central Ethiopia, women and community members indicated that the lack of privacy was one of the main reasons why women would not give birth in health facilities [15]. Given the importance of privacy, the WHO manual for managing complications in pregnancy and childbirth and the International Federation of Gynecology and Obstetrics (FIGO) have emphasised the need for privacy during childbirth and postpartum [35, 36]. Thus it is important that health facilities are supported to provide privacy for women to promote their dignity.

### Overall satisfaction with quality of care during childbirth

Interestingly, despite the lack of privacy, our study found high satisfaction with quality of care in both the survey and FGDs. Women’s satisfaction was based on the fact that some midwives were caring, respectful and supportive; that is, patient, empathetic, and would not shout or insult. Some midwives were available at all times to provide services and would make follow-up visits to women’s homes after childbirth. Findings from the Ghana demography and health survey are in line with our finding; 79% of women insured under the National Health Insurance Scheme were satisfied with the services received in a health facility [37]. However, the reasons for the satisfaction was different from that found for our study; the women were satisfied due to short waiting times for test results (56%) and cleanliness of health facilities (92%) [37]. Equally, a study in public health facilities in Gamo Gofa Zone, Southwest Ethiopia showed that 79.1% of women were happy with the overall services received during childbirth [38].

Our findings are not consistent with results from other studies in Ghana showing women dissatisfaction with the quality of care due to disrespect, abuse, abandonment and discrimination by health providers during childbirth [39, 40]. Given that health providers were not satisfied with the quality of health care in our study, probably women’s overall satisfaction might be attributed to the fact that the women had never experienced quality care previously. Without the opportunity to make comparisons or know what was possible, women might not know that a better quality of care existed, especially on attitudes and behaviours of providers. The dissatisfaction among health providers is due to the challenges found in the health system which affected the provision of quality care. Health providers are better placed to understand what constituted quality care, compared to the women and hence their expression of dissatisfaction.

### Strengths and limitations

Combining findings from surveys of facilities and women, FGDs, and IDIs, the study has demonstrated the unavailability of basic essential inputs like water, electricity, emergency transport and privacy in health facilities. It also showed the inadequacy of basic drugs, supplies, and equipment. This is one strength of this study. However, the high level of satisfaction reported in our study might be as a result of the halo effect. The joy of a successful live birth would not permit women to immediately express dissatisfaction with care received [41, 42]. Besides, our study measured perceived quality of care from the perspective of women and health providers. Although perceptions are important determinants for the utilisation of health services, measuring technical quality of care might have given different findings, which is beyond the scope of this paper. Hence readers should take note when reviewing this paper.

## Conclusion

There is lack of and inadequacy of basic essential inputs including clean water, electricity, emergency transport, drugs, supplies, equipment as well as privacy. While women were satisfied with care, health providers were not. Government needs to dedicate more resources to the provision of essential inputs, especially for CHPS compounds providing maternal health services. In addition, the district and sub-district health management committees should endeavour to play an active role in the management of health facilities to ensure efficiency and accountability. These measures would improve the provision of quality services and increase the use of skilled attendance for childbirth, leading to the achievement of universal health coverage and the sustainable development goals.

## Additional files

Additional file 1: Structured questionnaire (survey). Description of data: Structured questionnaire for interviews with women. (DOCX 25 kb)

Additional file 2: Survey of health facilities. Description of data: Checklist for health facility survey. (DOCX 19 kb)

Additional file 3: In-depth interviews. Description of data: Interview guide for in-depth interviews with health providers. (DOCX 16 kb)

Additional file 4: Focus group discussions**.** Description of data: Interview guide for focus group discussions with women. (DOCX 28 kb)

## Abbreviations

(CHPS): Community-based Health Planning and Services
FGDs: Focus groups discussions
IDIs: In-depth interviews
WASH: Water, Sanitation and Hygiene
WHO: World Health Organization

## References

[1] Essendi H, Johnson FA, Madise N, Matthews Z, Falkingham J, Bahaj AS, James P, Blunden L (2015). Infrastructural challenges to better health in maternity facilities in rural Kenya: community and healthworker perceptions. Reprod Health.

[2] Singh A (2016). Supply-side barriers to maternal health care utilization at health sub-centers in India. PeerJ.

[3] Adair-Rohani H, Zukor K, Bonjour S, Wilburn S, Kuesel AC, Hebert R, Fletcher ER (2013). Limited electricity access in health facilities of sub-Saharan Africa: a systematic review of data on electricity access, sources, and reliability. Glob Health Sci Pract.

[4] WHO (2015). Access to modern energy services for health facilities in resource-constrained settings: a review of status, significance, challenges and Measurement.

[5] WHO (2015). Water, sanitation and hygiene in health care facilities – urgent needs and actions: meeting report.

[6] WHO (2014). Meeting the fundamental need for water, sanitation and hygiene services in health care facilities: Meeting report.

[7] WHO (2015). Water, sanitation and hygiene in health care facilities: status in low- and middle-income countries and way forward.

[8] WHO (2016). Standards for improving quality of maternal and newborn care in health facilities.

[9] Hsia RY, Mbembati NA, Macfarlane S, Kruk ME (2012). Access to emergency and surgical care in sub-Saharan Africa: the infrastructure gap. Health Policy Plan.

[10] Khatri RB, Dangi TP, Gautam R, Shrestha KN, Homer CSE (2017). Barriers to utilization of childbirth services of a rural birthing center in Nepal: a qualitative study. PLoS One.

[11] Mkoka DA, Goicolea I, Kiwara A, Mwangu M, Hurtig A-K (2014). Availability of drugs and medical supplies for emergency obstetric care: experience of health facility managers in a rural district of Tanzania. BMC Pregnancy and Childbirth.

[12] Kanyangarara M, Munos MK, Walker N (2017). Quality of antenatal care service provision in health facilities across sub-Saharan Africa: evidence from nationally representative health facility assessments. J Glob Health.

[13] Diamond-Smith N, Sudhinaraset M, Montagu D (2016). Clinical and perceived quality of care for maternal, neonatal and antenatal care in Kenya and Namibia: the service provision assessment. Reprod Health.

[14] Banchani E, Tenkorang E (2014). Implementation challenges of maternal health care in Ghana: the case of health care providers in the tamale Metropolis. BMC Health Serv Res.

[15] Roro MA, Hassen EM, Lemma AM, Gebreyesus SH, Afework MF (2014). Why do women not deliver in health facilities: a qualitative study of the community perspectives in south Central Ethiopia?. BMC Research Notes.

[16] WHO (2010). Monitoring the building blocks of health systems: a handbook of indicators and their measurement strategies.

[17] Dalinjong PA, Wang AY, Homer CSE (2017). The operations of the free maternal care policy and out of pocket payments during childbirth in rural northern Ghana. Heal Econ Rev.

[18] Dalinjong PA, Wang AY, Homer CSE (2018). Has the free maternal health policy eliminated out of pocket payments for maternal health services? Views of women, health providers and insurance managers in northern Ghana. PLoS One.

[19] GSS (2014). 2010 Population and Housing Census: District Analytical Report; Kassena-Nankana East Municipality.

[20] GHS (2013). Upper East Regional Health Services 2013 Annu Rep.

[21] Nyonator FK, Awoonor-Williams KJ, Phllips JF, Tanya JC, Miller RA (2005). The Ghana community-based health planning and services initiative for scaling up service delivery innovation. Health Policy Plan.

[22] Creswell JW (2014). Research design: qualitative, quantitative and mixed methods approaches.

[23] Gorstein J, Sullivan KM, Parvanta I, Begin F (2007). Indicators and methods for cross-sectional surveys of vitamin and mineral status of populations. The micronutrient initiative (Ottawa) and the Centers for Disease Control and Prevention (Atlanta).

[24] WHO (2008). Toolkit on monitoring health systems strengthening: Service delivery.

[25] Mbonye AK, Asimwe JB (2010). Factors associated with skilled attendance at delivery in Uganda: results from a national health facility survey. Int J Adolesc Med Health.

[26] Nnebue CC, Ebenebe UE, Adogu POU, Adinma ED, Ifeadike CO, Nwabueze AS (2014). Adequacy of resources for provision of maternal health services at the primary health care level in Nnewi, Nigeria. Niger Med J.

[27] UNICEF (2011). Emergency Obstetric and Neonatal Care (EmONC) Needs Assessment in Selected Health Facilities in NEZ, Puntland.

[28] Atuoye KN, Dixon J, Rishworth A, Galaa SZ, Boamah SA, Luginaah I (2015). Can she make it? Transportation barriers to accessing maternal and child health care services in rural Ghana. BMC Health Serv Res.

[29] Vidler M, Ramadurg U, Charantimath U, Katageri G, Karadiguddi C, Sawchuck D, Qureshi R, Dharamsi S, Joshi A, von Dadelszen P (2016). Utilization of maternal health care services and their determinants in Karnataka state, India. Reproductive Health.

[30] Jammeh A, Sundby J, Vangen S (2011). Barriers to emergency obstetric Care Services in Perinatal Deaths in rural Gambia: a qualitative in-depth interview study. ISRN Obstet Gynecol.

[31] Kitui J, Lewis S, Davey G (2013). Factors influencing place of delivery for women in Kenya: an analysis of the Kenya demographic and health survey, 2008/2009. BMC Pregnancy Childbirth.

[32] Bohren MA, Vogel JP, Hunter EC, Lutsiv O, Makh SK, Souza JP, Aguiar C, Saraiva Coneglian F, Diniz ALA, Tunçalp Ö (2015). The mistreatment of women during childbirth in health facilities globally: a mixed-methods systematic review. PLoS Med.

[33] Ishola F, Owolabi O, Filippi V (2017). Disrespect and abuse of women during childbirth in Nigeria: a systematic review. PLoS One.

[34] Lothian JA (2004). Do not disturb: the importance of privacy in labor. J Perinat Educ.

[35] WHO (2017). Managing complications in pregnancy and childbirth: a guide for midwives and doctors in.

[36] FIGO (2015). Mother-baby friendly birthing facilities. Int J Gynecol Obstet.

[37] GSS, GHS, ICF (2015). Ghana demographic and health survey 2014.

[38] Tesfaye R, Worku A, Godana W, Lindtjorn B (2016). Client satisfaction with delivery care service and associated factors in the public health facilities of Gamo Gofa zone, Southwest Ethiopia: in a resource limited setting. Obstet Gynecol Int.

[39] Moyer CA, Adongo PB, Aborigo RA, Hodgson A, Engmann CM (2014). 'They treat you like you are not a human being': maltreatment during labour and delivery in rural northern Ghana. Midwifery.

[40] D'Ambruoso L, Abbey M, Hussein J (2005). Please understand when I cry out in pain: women's accounts of maternity services during labour and delivery in Ghana. BMC Public Health.

[41] Paudel YR, Mehata S, Paudel D, Dariang M, Aryal KK, Poudel P, King S, Barnett S (2015). Women's satisfaction of maternity Care in Nepal and its Correlation with intended future utilization. Int J Reprod Med.

[42] Seguin L, Therrien R, Champagne F, Larouche D (1989). The components of Women's satisfaction with maternity care. Birth.

