# Supplementary material for: Are health facilities well equipped to provide basic quality childbirth services under the free maternal health policy? Findings from rural Northern Ghana
Source: BMC Health Serv Res. 2018 Dec 12;18:959. doi: 10.1186/s12913-018-3787-1 (PMC6292018; doi:10.1186/s12913-018-3787-1)
Supplement: Supplementary file 1 — Structured questionnaire (survey). Description of data: Structured questionnaire for interviews with women. (DOCX 25 kb) [file 12913_2018_3787_MOESM1_ESM.docx]

## Structured questionnaire for interviews with women

Date of interview:

| **Socio-demographic background** | | | |
| --- | --- | --- | --- |
| Age | Occupation | | Highest educational level |
| (1)<20 | (1)Unemployed | | (1)No formal education |
| (2)20-24 | (2)Trader | | (2)Basic education |
| (3)25-29 | (3)Farmer | | (3)Secondary/technical education |
| (4)30-39 | (4)Public/civil servant | | (4)Tertiary education |
| (5)40+ | (5)Student | | (5)Other (specify)…………… |
|  | (6) Other (specify)………… | |  |
|  | | | |
| Marital status | Religious background | | Ethnicity |
| (1)Single | (1)Traditional | | (1)Kasem |
| (2)Married | (2)Catholic | | (2)Nakam |
| (3)Divorced | (3)Protestant | | (3)Other (specify)…………………….. |
| (4)Other (specify)……………… | (4)Muslim | |  |
|  | (5)Other (specify)…………………. | |  |
|  | | | |
| Number of births | Health insurance status | |  |
| (1)1 | (1)Insured | |  |
| (2)2 | (2)Uninsured | |  |
| (3)3 |  | |  |
| (4)4 or more |  | |  |
|  | | | |
| **Section A: Access to maternal health services during pregnancy** | | | |
| (1)Did you visit any facility for maternal health services during your last pregnancy? | | (2)What type of facility did you visit? | |
| (1)Yes | | (1)Community-based health planning and services (CHPS) | |
| (2)No | | (2)Public clinic | |
| (8)NA | | (3)Private clinic | |
| (9)Don’t know | | (4)Health centre | |
|  | | (5)District hospital | |
|  | | (6)Other (specify) | |
|  | | (8)NA | |
|  | | (9)Don’t know | |
|  | | | |
| (3)How much time does it take to reach that facility? | | (4)What do you think of the time it takes to reach the facility? | |
| (1)No travelling needed | | (1)Very short | |
| (2)Below 30 minutes | | (2)Short | |
| (3)30 minutes – 1 hour | | (3)Normal | |
| (4)1 hour – 2 hours | | (4)Long | |
| (5)More than 2 hours | | (5)Very long | |
| (6)Other (specify) | | (8)NA | |
| (8)NA | | (9)Don’t know | |
| (9)Don’t know | |  | |
|  | | | |
| (5)What do you think of the distance it takes to reach the facility? | | (6)What means of transport did you use to reach the facility? | |
| (1)Very near | | (1)Walk | |
| (2)Near | | (2)Bicycle | |
| (3)Normal | | (3)Motorbike | |
| (4)Far | | (4)Public transport | |
| (5)Very far | | (5)Private car | |
| (8)NA | | (6) Other (specify) | |
| (9)Don’t know | | (8)NA | |
|  | | | |
| (7)Does the opening hours of the facility suits your time? | | (8)What do you think about the waiting time at the facility? | |
| (1)Yes | | (1)Very short | |
| (2)No | | (2)Short | |
| (8)NA | | (3)Normal | |
| (9)Don’t know | | (4)Long | |
|  | | (5)Very long | |
|  | | (8)NA | |
|  | | (9)Don’t know | |
|  | | | |
| (9)Does the facility have a proper waiting area? | | (10)How is the cleanliness of the facility? | |
| (1)Yes | | (1)Very clean | |
| (2)No | | (2)Clean | |
| (8)NA | | (3)Normal | |
| (9)Don’t know | | (4)Dirty | |
|  | | (5)Very dirty | |
|  | | (8)NA | |
|  | | (9)Don’t know | |
|  | | | |
| (11)What do you think about the friendliness of the staff towards you? | | (12)What do you think about respectfulness of the staff towards you? | |
| (1)Very friendly | | (1)Very respectful | |
| (2)Friendly | | (2)Respectful | |
| (3)Normal | | (3)Normal | |
| (4)Unfriendly | | (4)Disrespectful | |
| (5)Very unfriendly | | (4)Very disrespectful | |
| (8)NA | | (8)NA | |
| (9)Don’t know | | (9)Don’t know | |
|  | | | |
| (13)Do you think there is privacy provided in the facility? | | (14)What health services did you receive when you visited the facility during your pregnancy? (multiple response) | |
| (1)Yes | | (1)Physical examination (including weight, blood pressure, heart rate) | |
| (2)No | | (2)Gynaecological examination | |
| (8)NA | | (3)Ultrasound | |
| (9)Don’t know | | (4)HIV/STD testing | |
|  | | (5)Blood tests | |
|  | | (6)Nutritional supplements | |
|  | | (7)Tetanus vaccine | |
|  | | (8)Other (specify) | |
|  | | (88)NA | |
|  | | (9)Don’t know | |
|  | | | |
| (15)Were any complications detected during your pregnancy? | | (16)If yes, were you referred to another facility for treatment? | |
| (1)Yes | | (1)Yes | |
| (2)No | | (2)No | |
| (8)NA | | (8)NA | |
| (9)Don’t know | | (9)Don’t know | |
|  | | | |
| (17)Does the health insurance cover all expenses in the primary facility? | | (18)If no, what expenses does the health insurance not cover? (multiple expenses) **(state total amount if paid by client)** | |
| (1)Yes | | (1)Folder fee (GH¢………..) | |
| (2)No | | (2)Consultation (GH¢………..) | |
| (8)NA | | (3)Laboratory test (GH¢………..) | |
| (9)Don’t know | | (4)Drugs (GH¢………..) | |
|  | | (5)Blood (GH¢………..) | |
|  | | (6)Feeding (GH¢………..) | |
|  | | (7)Hospitalisation (GH¢………..) | |
|  | | (8)Transport (GH¢………..) | |
|  | | (9)Other (specify (GH¢………..) | |
|  | | (88)NA | |
|  | | (99)Don’t know | |
|  | | | |
| (19)How did you pay for the expenditure in question 18? | | (20)Did you pay any unofficial fee for maternal health services during your pregnancy? | |
| (1)Used savings | | (1)Yes | |
| (2)Borrowed money | | (2)No | |
| (3)Sold assets | | (8)NA | |
| (4)Other (specify) | | (9)Don’t know | |
| (8)NA | |  | |
| (9)Don’t know | |  | |
|  | | | |
| (21)What was the unofficial fee paid for? | | (22)Was it demanded or did you pay it on your own? | |
| (1)Extra services | | (1)Demanded | |
| (2)Extra drugs | | (2)Paid on my own | |
| (3)Other (specify) | | (8)NA | |
| (8)NA | | (9)Don’t know | |
| (9)Don’t know | |  | |
|  | | | |
| (23)How many times did you visit that facility during your pregnancy? | | (24)How do you think access to maternal health services during pregnancy can be improved? | |
| (1)1 to 3 visits | | (1)Reduced travel distance & time | |
| (2)More than 3 visits | | (2)Reduced waiting time | |
| (8)NA | | (3)Reduced cost | |
| (9)Don’t know | | (4)Good providers relations | |
|  | | (5)Providing qualified staff | |
|  | | (6)Improving drugs supplies | |
|  | | (7)Other (specify) | |
|  | | (8)NA | |
|  | | (9)Don’t know | |
|  | | | |
| (25)What is your overall satisfaction for maternal health service received during your last pregnancy? | | (26)What was the primary reason for not using a formal health facility for giving birth during your last pregnancy? **(For women who gave birth at home).** | |
| (1)Very satisfied | | (1)Long distance | |
| (2)Satisfied | | (2)Birthing services not available | |
| (3)Normal | | (3)No qualified staff | |
| (4)Dissatisfied | | (4)Drugs not available | |
| (5)Very dissatisfied | | (5)Not satisfied with services received) | |
| (8)NA | | (6)Bad provider relations | |
| (9)Don’t know | | (7)High treatment cost | |
|  | | (8)Other (specify) | |
|  | | (88)NA | |
|  | | (9)Don’t know | |
|  | | | |
| **Section B: Access to maternal health services during childbirth**  **(Skip section B and move to section C for women who gave birth at home)** | | | |
| (27)Did you give birth in a facility different from the one you used during your last pregnancy**? (If response is “No”, skip to Q 39)** | | (28)If yes, why did you change a facility? | |
| (1)Yes | | (1)Was referred | |
| (2)No | | (2)Birthing services available | |
| (8)NA | | (3)Qualified staff available | |
| (9)Don’t know | | (4)Drugs available | |
|  | | (5)Quality services available | |
|  | | (6)Good provider relations | |
|  | | (7)Low treatment cost | |
|  | | (8)Short distance & time | |
|  | | (9)Other (specify) | |
|  | | (88)NA | |
|  | | (99)Don’t know | |
|  | | | |
| (29)What type of facility did you give birth in? | | (30)How much time does it take to reach that facility? | |
| (1)Community-based health and planning services (CHPS) | | (1)No travelling needed | |
| (2)Public clinic | | (2)Below 30 minutes | |
| (3)Private clinic | | (3)30 minutes – 1 hour | |
| (4)Health centre | | (4)1 hour – 2 hours | |
| (5)District hospital | | (5)More than 2 hours | |
| (6)Other (specify) | | (6)Other (specify) | |
| (8)NA | | (8)NA | |
| (9)Don’t know | | (9)Don’t know | |
|  | | | |
| (31)What do you think of the time it takes to reach the facility? | | (32)What do you think of the distance it takes to reach the facility? | |
| (1)Very short | | (1)Very near | |
| (2)Short | | (2)Near | |
| (3)Normal | | (3)Normal | |
| (4)Long | | (4)Far | |
| (5)Very long | | (5)Very far | |
| (8)NA | | (8)NA | |
| (9)Don’t know | | (9)Don’t know | |
|  | | | |
| (33)What means of transport did you use to reach the facility? | | (34)Does the opening hours of the facility suits your time? | |
| (1)Walk | | (1)Yes | |
| (2)Bicycle | | (2)No | |
| (3)Motorbike | | (3)NA | |
| (4)Public transport | | (4)Don’t know | |
| (5)Private car | |  | |
| (6)NA | |  | |
|  | | | |
| (35)How is the cleanliness of the facility? | | (36)What do you think about the friendliness of the staff towards you? | |
| (1)Very clean | | (1)Very friendly | |
| (2)Clean | | (2)Friendly | |
| (3)Normal | | (3)Normal | |
| (4)Dirty | | (3)Unfriendly | |
| (5)Very dirty | | (4)Very unfriendly | |
| (8)NA | | (8)NA | |
| (9)Don’t know | | (9)Don’t know | |
|  | | | |
| (37)What do you think about respectfulness of the staff towards you? | | (38)Do you think there is privacy provided in the facility? | |
| (1)Very respectful | | (1)Yes | |
| (2)Respectful | | (2)No | |
| (3)Normal | | (8)NA | |
| (4)Disrespectful | | (9)Don’t know | |
| (5)Very Disrespectful | |  | |
| (8)NA | |  | |
| (9)Don’t know | |  | |
|  | | | |
| (39)During the birth of your child, which type of health worker attended to you? | | (40)Were any complications detected during the birth of your child? | |
| (1)Doctor | | (1)Yes | |
| (2)Midwife | | (2)No | |
| (3)Nurse | | (8)NA | |
| (4)Other (specify) | | (9)Don’t know | |
| (8)NA | |  | |
| (9)Don’t know | |  | |
|  | | | |
| (41)If yes, were you referred to a higher facility for treatment? | | (42)Were all expenses covered by the health insurance during the birth of your child? | |
| (1)Yes | | (1)Yes | |
| (2)No | | (2)No | |
| (8)NA | | (8)NA | |
| (9)Don’t know | | (9)Don’t know | |
|  | | | |
| (43)If no, what expenses were not covered? (multiple expenses) **(state total amount if paid by client)** | | (44)How did you pay for the expenditure in question 43? | |
| (1)Folder fee (GH¢………..) | | (1)Used savings | |
| (2)Consultation (GH¢………..) | | (2)Borrowed money | |
| (3)Laboratory test (GH¢………..) | | (3)Sold assets | |
| (4)Drugs (GH¢………..) | | (4)Other (specify) | |
| (5)Blood (GH¢………..) | | (8)NA | |
| (5)Feeding (GH¢………..) | | (9)Don’t know | |
| (6)Hospitalization (GH¢………..) | |  | |
| (7)Transport (GH¢………..) | |  | |
| (8)Other (specify) (GH¢………..) | |  | |
| (88)NA | |  | |
| (9)Don’t know | |  | |
|  | | | |
| (45)Did you pay any unofficial fee during the birth of your child? | | (46)What was the unofficial fee paid for? | |
| (1)Yes | | (1)Extra services | |
| (2)No | | (2)Extra drugs | |
| (8)NA | | (3)Other (specify) | |
| (9)Don’t know | | (8)NA | |
|  | | (9)Don’t know | |
|  | | | |
| (47)Was it demanded or did you pay it on your own? | | (48)How do you think access to maternal health services during child birth can be improved? | |
| (1)Demanded | | (1)Reduced travel distance & time | |
| (2)Paid on my own | | (2)Reduced waiting time | |
| (8)NA | | (3)Reduced cost | |
| (9)Don’t know | | (4)Good provider relations | |
|  | | (5)Providing qualified staff | |
|  | | (6)Improving drugs supplies | |
|  | | (7)Other (specify) | |
|  | | (8)NA | |
|  | | (9)Don’t know | |
|  | | | |
| (49)What is your overall satisfaction for maternal health service received during the birth of your child? | |  | |
| (1)Very satisfied | |  | |
| (2)Satisfied | |  | |
| (3)Normal | |  | |
| (4)Dissatisfied | |  | |
| (5)Very dissatisfied | |  | |
| (8)NA | |  | |
| (9)Don’t know | |  | |
| **Section C:Access to maternal health services after childbirth at home)**  **(For women who gave birth at home)** | | | |
| (50)Did you visit any facility after the birth of your child at home? | | (51)What type of facility did you visit? | |
| (1)Yes | | (1)Community-based health and planning services (CHPS) | |
| (2)No | | (2)Public clinic | |
| (8)NA | | (3)Private clinic | |
| (9)Don’t know | | (4)Health centre | |
|  | | (5)District hospital | |
|  | | (6)Other (specify) | |
|  | | (8)NA | |
|  | | (9)Don’t know | |
|  | | | |
| (52)How many times did you visit the facility after the birth of your child? | | (53)What health services did you receive when you visited the facility after your child’s birth? (multiple response) | |
| (1)1 to 2 visits | | (1)Physical examination | |
| (2)More than 2 visits | | (2)Counselling on breastfeeding | |
| (8)NA | | (3)Contraceptives | |
| (9)Don’t know | | (4)Blood test for anemia | |
|  | | (5)Nutritional supplements | |
|  | | (6)Information on warning signs of problems | |
|  | | (7)Other (specify) | |
|  | | (8)NA | |
|  | | (9)Don’t know | |
|  | | | |
| (54)Did you experience any problem after the birth of your child? | | (55)Were you referred to other facility? | |
| (1)Yes | | (1)Yes | |
| (2)No | | (2)No | |
| (8)NA | | (8)NA | |
| (9)Don’t know | | (9)Don’t know | |
