# Supplementary material for: Are health facilities well equipped to provide basic quality childbirth services under the free maternal health policy? Findings from rural Northern Ghana
Source: BMC Health Serv Res. 2018 Dec 12;18:959. doi: 10.1186/s12913-018-3787-1 (PMC6292018; doi:10.1186/s12913-018-3787-1)
Supplement: Supplementary file 2 — Survey of health facilities. Description of data: Checklist for health facility survey. (DOCX 19 kb) [file 12913_2018_3787_MOESM2_ESM.docx]

## Checklist for health facility survey

Date:

Name of health facility:

Type of health facility:

**Checklist for basic equipment, drugs and vaccines for maternal and child health services**

| **Basic delivery care** | | | | | |
| --- | --- | --- | --- | --- | --- |
| Staff & training | Y/N | Drugs & vaccines | Qty available | Qty req. | Reasons for shortfall /unavailability |
| Guidelines available? |  | Antibiotic eye ointment (newborn) |  |  |  |
| Provider trained (midwife)? |  | Skin disinfectant |  |  |  |
| Equipment |  | Injectable oxytoxic/ergometrine |  |  |  |
| Emergency transport |  | Oral antibiotic |  |  |  |
| Gloves |  | Anticonvulsant |  |  |  |
| Delivery bed |  | Magnesium sulphate |  |  |  |
| Partograph |  | Injectable antibiotic |  |  |  |
| Examination light |  |  |  |  |  |
| Scissors & blade |  |  |  |  |  |
| Cord clamp |  |  |  |  |  |
| Suction apparatus |  |  |  |  |  |
| Needles & syringes |  |  |  |  |  |
| IV Solution & infusion set |  |  |  |  |  |
| Suture material & needle holder |  |  |  |  |  |
| Forcept |  |  |  |  |  |
| Speculum |  |  |  |  |  |
|  |  |  |  |  |  |
|  |  |  |  |  |  |
|  |  |  |  |  |  |
| **Basic child health services** | | | | | |
| Staff & training | Y/N | Drugs & vaccines | Qty available | Qty req. | Reasons for shortfall /unavailability |
| Guidelines available? |  | ORS packet |  |  |  |
| Provider trained (at least 1 in last 2 years)? |  | Vitamin A |  |  |  |
| Equipment |  | Amoxicillin |  |  |  |
| Refrigerator |  | Cotrimoxazol |  |  |  |
| Child weighing scale |  | Paracetamol |  |  |  |
| Thermometer |  | Iron tablets |  |  |  |
| Stethoscope |  | Me-/albendazole |  |  |  |
|  |  |  |  |  |  |
|  |  |  |  |  |  |
|  |  |  |  |  |  |
|  |  |  |  |  |  |
|  |  |  |  |  |  |
| **Basic child immunisations** | | | | | |
| Staff & training | Y/N | Drugs & vaccines | Qty available | Qty req. | Reasons for shortfall /unavailability |
| Guidelines available? |  | Measles |  |  |  |
| Provider trained? |  | DPT-HB |  |  |  |
| Equipment |  | Polio |  |  |  |
| Health cards |  | BCG |  |  |  |
| Tally sheets & register |  | Yellow Fever |  |  |  |
| Needles & syringes |  | PCV |  |  |  |
| Cold box & ice packs |  | Rotarex |  |  |  |
| Soap & water |  |  |  |  |  |
| Sharps box |  |  |  |  |  |
