# Supplementary material for: Are health facilities well equipped to provide basic quality childbirth services under the free maternal health policy? Findings from rural Northern Ghana
Source: BMC Health Serv Res. 2018 Dec 12;18:959. doi: 10.1186/s12913-018-3787-1 (PMC6292018; doi:10.1186/s12913-018-3787-1)
Supplement: Supplementary file 3 — In-depth interviews. Description of data: Interview guide for in-depth interviews with health providers. (DOCX 16 kb) [file 12913_2018_3787_MOESM3_ESM.docx]

## Interview guide for in-depth interviews with health providers

Date of interview: Type of facility:

Position of interviewee:

Number of years of practice:

What is the opening and closing time for this facility?

What are the health services provided by this facility?

What is the free maternal health policy?

What services are covered under the free maternal health policy for pregnant women?

What services are not covered under the free maternal health policy?

What do you think of the capacity of this facility to provide services under the free maternal health policy? In terms of staff, infrastructure, equipment, drugs and supplies, emergency transport, etc.

What costs are not covered under the free maternal health policy?

What will make pregnant women to be happy/not happy with health services provided from this facility?

Do you think the use of maternal health services is affected by a) culture b) religion c) sex of the provider? How?

How satisfied are you with the quality of maternal health services provided in this facility? Why?

How motivated are you in this era of the free maternal health policy?

As a provider, what challenges confront the provision of maternal health service?

How do you think these challenges can be solved for providers to be able to provide services?

What are the challenges faced by women in accessing maternal health service?

How do you think these challenges can be solved for women to be able to access maternal health services?
