# Supplementary material for: Are health facilities well equipped to provide basic quality childbirth services under the free maternal health policy? Findings from rural Northern Ghana
Source: BMC Health Serv Res. 2018 Dec 12;18:959. doi: 10.1186/s12913-018-3787-1 (PMC6292018; doi:10.1186/s12913-018-3787-1)
Supplement: Supplementary file 4 — Focus group discussions. Description of data: Interview guide for focus group discussions with women. (DOCX 28 kb) [file 12913_2018_3787_MOESM4_ESM.docx]

**Interview guide for focus group discussions with women**

**(For both women who gave birth at health facilities and at home)**

Are health facilities available in this community?

Where do you go for health services?

What do you think of the time/distance it takes to reach facilities providing maternal health services?

What do you think about the attitude of the staff towards you/pregnant women? In terms of friendliness, respectfulness, etc.

Does culture/religion/sex of the provider affect your use of maternal health services? How?

Have you heard of the free maternal health policy?

What is the free maternal health policy?

What health services are covered under the free maternal health policy?

What health services are not covered under the free maternal health policy?

What costs are covered by the free maternal health policy? (Folder fee, consultation, drugs, laboratory tests, admissions, blood, oxygen, feeding on admission)

What costs are not covered by the free maternal health policy? (Feeding, transport, drugs and services not in the essential drugs list).

**(For women who gave birth in health facilities)**

What encouraged you to give birth in a health facility during your last pregnancy?

What is your overall satisfaction for maternal health service received during the birth of your child?

Will you recommend this facility to a relative/friend in the future? Why?

How do you think access to maternal health services during child birth can be improved?

**(For women who gave birth at home)**

Have you given birth in a health facility before?

What made you not to give birth in a health facility during your last pregnancy?

For subsequent pregnancies, what place of birth will you use? Why?

What can be done to encourage pregnant women to give birth in health facilities?
